# Expression of Concern: Role of microRNA-15a-5p in the atherosclerotic inflammatory response and arterial injury improvement of diabetic by targeting FASN

**DOI:** 10.1042/BSR-20181852_EOC

**Published:** 2021-03-31

**Keywords:** Arterial injury, Atherosclerosis, Atherosclerotic inflammation, Diabetes, miR-15a-5p

The Editorial Office has been made aware of potential issues surrounding the scientific validity of this paper, hence has issued an expression of concern to notify readers whilst the Editorial Office investigates.

